# Inducing the Alternative Oxidase Forms Part of the Molecular Strategy of Anoxic Survival in Freshwater Bivalves

**DOI:** 10.3389/fphys.2018.00100

**Published:** 2018-02-23

**Authors:** Maria S. Yusseppone, Iara Rocchetta, Sebastian E. Sabatini, Carlos M. Luquet, Maria del Carmen Ríos de Molina, Christoph Held, Doris Abele

**Affiliations:** ^1^Laboratorio de Enzimología, Estrés y Metabolismo, INQUIBICEN, Departamento de Química Biológica, Facultad de Ciencias Exactas y Naturales, Universidad de Buenos Aires, Consejo Nacional de Investigaciones Científicas y Técnicas, Buenos Aires, Argentina; ^2^Laboratorio de Ecotoxicología Acuática, INIBIOMA, Universidad Nacional del Comahue, Consejo Nacional de Investigaciones Científicas y Técnicas, Junín de los Andes, Argentina; ^3^Department of Functional Ecology, Alfred Wegener Institute, Helmholtz Centre for Polar and Marine Research, Bremerhaven, Germany

**Keywords:** hypoxia, oxidative stress, alternative oxidase, mitochondrial electron transport, anaerobiosis, *Diplodon chilensis*

## Abstract

Hypoxia in freshwater ecosystems is spreading as a consequence of global change, including pollution and eutrophication. In the Patagonian Andes, a decline in precipitation causes reduced lake water volumes and stagnant conditions that limit oxygen transport and exacerbate hypoxia below the upper mixed layer. We analyzed the molecular and biochemical response of the North Patagonian bivalve *Diplodon chilensis* after 10 days of experimental anoxia (<0.2 mg O_2_/L), hypoxia (2 mg O_2_/L), and normoxia (9 mg O_2_/L). Specifically, we investigated the expression of an alternative oxidase (AOX) pathway assumed to shortcut the regular mitochondrial electron transport system (ETS) during metabolic rate depression (MRD) in hypoxia-tolerant invertebrates. Whereas, the AOX system was strongly upregulated during anoxia in gills, ETS activities and energy mobilization decreased [less transcription of glycogen phosphorylase (GlyP) and succinate dehydrogenase (SDH) in gills and mantle]. Accumulation of succinate and induction of malate dehydrogenase (MDH) activity could indicate activation of anaerobic mitochondrial pathways to support anoxic survival in *D. chilensis*. Oxidative stress [protein carbonylation, glutathione peroxidase (GPx) expression] and apoptotic intensity (caspase 3/7 activity) decreased, whereas an unfolded protein response (HSP90) was induced under anoxia. This is the first clear evidence of the concerted regulation of the AOX and ETS genes in a hypoxia-tolerant freshwater bivalve and yet another example that exposure to hypoxia and anoxia is not necessarily accompanied by oxidative stress in hypoxia-tolerant mollusks.

## Introduction

Hypoxic conditions defined as oxygen concentrations of <2 mg/L have become ever more widespread in marine and freshwater systems during the twentieth century (Hawley et al., 2006; Diaz and Rosenberg, 2008; Levin et al., 2009). Lakes can turn hypoxic as a result of nutrient overload in farming areas, especially when water exchange is reduced and the water becomes stagnant. As a consequence of climate change, surface temperatures in lakes and ponds increase whereas precipitation rates decrease (SADSN, 2014). This stabilizes stratification and promotes oxygen depletion below the thermocline (Paerl et al., 1998; Hawley et al., 2006). Hypoxia provokes behavioral responses (swimming and surfacing in motile animals) and metabolic reorganization in aquatic invertebrates to survive a hypoxic episode until water masses are mixed or exchanged and oxygen returns. In lakes where hypoxia is common under winter ice cover or during summer stratification, some invertebrate and fish species are of astonishing hypoxia tolerance and survive days and even months at very low dissolved oxygen levels (e.g., the crucian carp is famous for its hypoxic tolerance) (Bickler and Buck, 2007).

Most studies to investigate the metabolic pathways involved in hypoxic survival in invertebrates have so far been focused on marine organisms, and bivalves have been recognized as one of the most hypoxia and anoxia tolerant groups among marine benthos fauna (Theede, 1973; Zurburg and Kluytmans, 1980; Grieshaber et al., 1994; Larade and Storey, 2002). Fewer studies have analyzed the response of fresh water bivalves to diminishing oxygen levels and the anaerobic pathways they employ (Dietz, 1974; Gäde and Wilps, 1975; Sheldon and Walker, 1989). Both groups of bivalves, fresh water and marine, survive hypoxia and anoxia by two basic strategies. As the environmental oxygen levels decline they rapidly reduce metabolic energy expenditures (measurable as difference in aerobic metabolic rate or heat production) by as much as 90% of normoxic rates (Abele et al., 2017). Furthermore, in the absence of oxygen, bivalves can mobilize energy reserves such as glycogen. In addition, marine bivalves, such as *Crassostrea gigas* (Collicutt and Hochachka, 1977) and *Mytilus edulis* (De Zwaan and Wijisman, 1976), maintain a larger pool of free amino acids for osmotic balance (De Zwaan and Wijisman, 1976) and can use aspartate to fuel ATP generating mitochondrial pathways (De Zwaan and Wijisman, 1976; Issani et al., 1995; Anestis et al., 2010), a special capacity of their “anaerobically functioning mitochondria” (van Hellemond et al., 2003). Many hypoxia tolerant species display a capacity for metabolic rate depression (MRD), which is rapidly induced by reversible phosphorylation, activating or deactivating glycolytic regulatory key enzymes, such as glycogen phosphorylase (GlyP), pyruvate kinase (PK), and phosphoenolpyruvate carboxykinase (PEPCK). In the state of MRD anaerobic intermediates of mitochondrial fermentation pathways that produce ATP in the absence of oxygen (succinate, as well as acetate and/or propionate) accumulate (for review see Storey and Storey, 1990).

Another peculiarity of bivalve mitochondria is the occurrence of the alternative oxidase (AOX) (van Hellemond et al., 2003). Insensitive to sulfide (and NO) inhibition, the AOX oxidizes ubiquinol and reduces O_2_ to water and hence maintains electron transport when cytochrome oxidase is inhibited under sulfidic conditions. Alternative oxidase has been detected biochemically and sequenced in several hypoxia tolerant species, most of them marine bivalves (McDonald et al., 2009), and has been ascribed several physiological functions. First of all, it protects anaerobic mitochondria (and their bearers) from respiratory poisoning by hydrogen sulfide. Secondly, it relaxes the electron transport system (ETS) by deviating electrons away from the classical phosphorylation sites at complexes III and IV (cytochrome oxidase), reducing oxygen without pumping protons across the inner mitochondrial membrane during metabolic shutdown. In so doing, AOX relaxes the inner membrane proton gradient and membrane potential (ΔΨm), theoretically lowering the reduction state of respiratory chain components such as ubiquinone and preventing them from autoxidizing during reoxygenation (summarized in Abele et al., 2017). Autoxidation of highly reduced intermediates would liberate detrimental reactive oxygen species (ROS) and cause an oxidative burst reaction as it typically occurs during hypoxia reoxygenation injury in mammalian brain and hypoxia sensitive species. In view of these potential oxidative stress hazards, the theory of preventive activation of antioxidant mechanisms during hypoxia has been developed (Welker et al., 2013). Although this hypothesis works well for several hypoxia tolerant animal models, bivalves with their hypoxia competent mitochondria may represent an exception. Several studies have failed to show hypoxic and/or anoxic induction of antioxidant gene expression or enzyme activities in marine bivalves (Strahl et al., 2011b in *Arctica islandica*, Rivera-Ingraham et al., 2013a in *M. edulis*, Sussarellu et al., 2012 in *C. gigas*). Philipp et al. (2012) found induction of antioxidants [superoxide dismutase (SOD) and catalase (CAT)] in concert with other stress genes upon 3.5 days exposure to experimental anoxia, but not under hypoxia exposure in gills of *A. islandica* from the Baltic Sea. However, the enzyme activities were lower (CAT) or unchanged (glutathione peroxidase, GPx) in anoxia compared to normoxic levels. Furthermore, no stress gene induction was detectable in North Sea *A. islandica* gills under the same exposure conditions. Thus, there is currently no unifying and simple concept of how antioxidants respond to hypoxia and anoxia in bivalves. Interestingly, 12–24 h of hypoxic exposure induced gene expression of the AOX in gills and digestive gland of the pacific oyster (*C. gigas*, Sussarellu et al., 2012), potentially as an alternative way of mitigating ROS formation during reoxygenation. This was our motivation to include the analysis of AOX gene expression in our study of the hypoxia/anoxia tolerance of *Diplodon chilensis* (Gray, 1828).

The unionid bivalve *D. chilensis* is an abundant colonizer of sandy or muddy grounds in lakes and rivers in southern Argentina and Chile. The high abundance and clearance capacity render *D. chilensis* important to the conservation of the oligotrophic environments of lakes and rivers in Patagonia (Ribeiro Guevara et al., 2005; Valdovinos and Pedreros, 2007). *D. chilensis* is of considerable hypoxia tolerance. Grandón et al. (2008) exposed individuals to experimental anoxia for 30 days with only 20% mortality resulting, which suggests these bivalves can survive in eutrophicated and hypoxic environments. On the other hand, population decline of *D. chilensis* during the last years has been associated with a decrease in water quality in Patagonian lakes (Valdovinos and Pedreros, 2007; Sabatini et al., 2011; Rocchetta et al., 2014).

In the present study, we analyzed the molecular and biochemical response of *D. chilensis* to 10 days of hypoxic or anoxic exposure. We combined the analysis of metabolic regulation at the level of gene expression and metabolic enzyme activities with the analysis of diverse antioxidant and stress parameters in gill and mantle. We further measured the induction of the AOX at gene expression level and assayed the activity of the mitochondrial ETSs in gill and mantle tissues. From this we derive a set of biomarkers that can be used to detect states of oxygen deficiency in Patagonian lakes where *Diplodon* is present.

## Materials and methods

### Animal collection and maintenance

Adult individuals of *D. chilensis* (74.98 ± 4.00 mm shell length) were collected at 8 m water depth by SCUBA diving from Paimún Lake (39°44.78′S 71°31.48′W), Patagonia, Argentina in April 2014. In these lakes the water temperature ranges from 17°C during spring and summer to around 10°C during autumn and winter. During the sampling month in autumn surface water temperature was 13.39 ± 0.15°C and dissolved oxygen was at 9.40 ± 0.30 mg/L in the water column above the muddy-sandy substrate). Live animals were shipped to the University of Buenos Aires (Facultad de Ciencias Exactas y Naturales) Argentina and maintained in aquaria at constant temperature of 10°C under normoxic conditions during 8 weeks of acclimation phase. Water quality was assessed weekly using a multiparameter analyzer (YSI Pro Plus Quatro Field Cable, YSI Inc., Yellow Springs, USA) for pH, temperature, and oxygen levels. Total ammonium and nitrate levels were measured spectrophotometrically (HACH 8155 and 8192). Mussels were fed three times per week with a culture of living microalgae *Scenedesmus vacuolatus* (10^6^ cells/mL).

Lanin National Park permissions were required for the sample collections (N° 815). The freshwater mussel *D. chilensis* is not a protected species and neither included in the Argentinian Council for Animal care procedures. The research conducted with *D. chilensis* has the approval of the Ethics Committee of the Facultad de Ciencias Exactas y Naturales, Universidad de Buenos Aires (Faculty of Exact and Natural Sciences, University of Buenos Aires).

### Experimental design

A pre-experiment was carried out to determine the number of days needed to see effects of anoxia on the ecophysiological capacities of *D. chilensis*. For this purpose, bivalves were exposed to normoxic and anoxic conditions (*n* = 12) in individual glass jars and performance was tested daily by checking the mantle and foot response to mechanical stimulation. Fifty percent of all animals exposed to anoxic conditions were unresponsive on day 13 of exposure, whereas no unresponsive individuals were observed in the normoxic treatment. Hence, we decided to expose animals for 10 days in the main experiment.

Mussels were randomly assigned to three treatments: anoxia (0.2 mg O_2_/L (0.5 kPa O_2_), hypoxia (2 mg O_2_/L (5 kPa O_2_), and normoxia (9 mg O_2_/L (21 kPa O_2_). In each treatment 10 bivalves were maintained for 10 days in individual glass jars (1 bivalve per jar) with 800 mL of dechlorinated tap water at 10°C in a stagnant water system. Hypoxic and anoxic conditions were obtained by bubbling nitrogen, and O_2_ concentration was monitored using an oxygen electrode (LT Lutron DO-5510, Lutron Electronics Inc., Taipei, Taiwan). Control jars were purged with air. Water quality was assessed as described above and incubation water was completely renewed every 5 days.

After 10 days of exposure to anoxia, hypoxia, and normoxia, mantle and foot retraction upon mechanical stimulation was checked to make sure all animals were alive. Bivalves were sacrificed by cutting through the adductor muscle, and gill and mantle were shock frozen in liquid nitrogen and stored at −80°C. A piece of each tissue was preserved in RNAlater (Sigma-Aldrich) for molecular biology analysis and stored at −20°C. Samples were shipped to the Alfred-Wegener-Institute for Polar and Marine Research (AWI), Bremerhaven, Germany, where they were stored at −80°C prior to analysis.

### Gene expression analysis

Forward and reverse primers sequences used for qPCR are shown in Table 1. Primer sequences (Table 1) were obtained from a transcriptome data set for *D. chilensis* (Rocchetta et al., in preparation) generated using Illumina technology in samples from two different populations (Paimún lake and Chimehuin river) to obtain ESTs from the most highly expressed genes. Functional annotation was performed using the Trinotate functional annotation suite version 07-08-2014 (Grabherr et al., 2011). Homology search was done against the Swiss-Prot database (Uniprot) with an e-value cut-off of 1e-9. The resulting sequences were translated to proteins using all six open-reading frames (ORF) using the invertebrate mitochondrial code (code 5: invertebrates, mitochondrial). The correct reading frame was checked using ORFfinder https://www.ncbi.nlm.nih.gov/orffinder/). Amino acid sequences were identified by blasting against NCBI (https://blast.ncbi.nlm.nih.gov/Blast.cgi) and the transcriptomes available by using CLC Main Workbench v6. Primers were designed also using CLC Main Workbench v6 considering CG or GC ratio >55%, a fragment length between 100 and 250 bp. Candidate primer pairs were double checked for primer dimers using PerlPrimer V1.1.21. Each primer pair was evaluated on the grounds of the absence of artifacts in their melting curve and their efficiency was assessed by serial template dilution (Table 1). The relative quantification of gene expression was carried out by the comparative CT method (delta Ct) using Q-Gene software (Joehanes and Nelson, 2008). The software Normfinder https://moma.dk/normfinder-software was used to find suitable reference genes for normalization. Amongst four reference gene candidates (Table 1), RPL8 was identified as the most suitable for gene normalization.

**Table 1:** Genes and primers used for qPCR analyses in *D. chilensis*.

**Category**	**Accession**	**Candidate gene**	**5′-3′ Forward primer**	**5′-3′ Reverse primer**	**Amplicon length (bp)**	**Primer efficiency**
Metabolism	MF771090	Pyruvate kinase (PK)	CTAGCATTGCCCAGACATC	CTGTGATTGGTGCTAGGTGC	113	1.14
	MF771093	Glycogen phosphorylase (GlyP)	CTGGTCTAGGAAATGGCGG	GTTCTTCTGTCTGCCACCC	112	1.2
	MF771092	Succinate dehydrogenase (SDH)	CTGCTTCTTGCCGATGTCTG	CCCATGGTTCTTGATGCTC	267	1.18
	MF771091	Citrate synthase (CS)	GATGTATGGAGGAATGCGAGG	CTGTGCTTGTGTAGGGATGTC	163	1.13
	MF771094	Alternative oxidase (AOX)	CCTGCACTACCATTCCTCC	CGTCTCGTCAATCATACCC	231	1.18
Defense system	MF695710	Glutathione synthetase (GSS)	GCTGGTAGTGTATGGAGGGAC	CGATGAAGACAGGAAGCTG	121	1.19
	MF771089	Glutathione peroxidase (GPX)	GGTCAATGGTCGATGCGTAG	GGGGAGGAGCTATTGAATGG	93	0.98
Stress response and damage cell	MF774028	HSP70	CTCCTGTTGTTGGATGTTGCC	GTTGTCTTTGGTCATGGCTC	148	1.1
	MF774029	HSP90	CCTTAATGCGCCTCTCTTCC	GATAGGACAGTTTGGTGTGGG	183	1.12
	MF774030	Laminin receptor	CAACCTGAAAAAGACCTGGG	GGGAGTCAGTATTGCACAAGG	270	1.07
Housekeeper	MF774031	Tubulin	GAACCATACAACGCCACC	GAAATGTAGACGTGGGAAGGG	216	1.18
	MF774032	Beta-Actin	CATTGTGGGAAGACCTAGGC	GATCTTCTCCATGTCGTCCC	117	1.09
	MF776702	EF1-alfa	GGACACGTAGACTCTGGAAAG	GTCTCAAACTTCCACAGGGC	156	1.12
	MF776703	RPL8	CTGTATGGAGGAGAAGGCTGG	GTTCATGGCCACACCTCTG	234	1.17

Mantle and gill samples preserved in RNAlater from the three experimental groups (normoxia, hypoxia, anoxia) were used to compare expression levels of selected genes [GPx, Glutathione synthetase (GSS), PK, Citrate synthase (CS), Succinate dehydrogenase (SDH), GlyP, AOX, HSP70, HSP90, Laminin receptor (LR), Tubulin, Beta-Actin, EF1-alfa, RPL8, Table 1]. Mantle and gill (*n* = 5) per treatment and tissue, except for normoxia-exposed bivalves in mantle (*n* = 4), were homogenized in RLT lysis buffer (Qiagen) and 1% β-mercaptoethanol (v/v) using a Precellys homogenizer (Precellys24, Bertin Technologies, France). Total RNA was extracted with a Qiagen RNeasy kit following the manufacturer's instructions. RNA quality and quantity were analyzed using a nanodrop spectrophotometer (ND-1000, Peqlab, USA) and cDNA was generated with the High capacity cDNA RT Kit (Applied Biosystems, USA). Gene expression levels were studied by real time PCR (q-PCR, Rotor-Gene Q, Qiagen). Cycling conditions were initial denaturing at 95°C for 5 min followed by 40 cycles at 95°C for 10 s, 55°C for 30 s, DNA fragments were melted from 65 to 90°C ramping in 0.5°C steps for each fluorescence measurement. Every sample was tested in double.

### Enzyme activity

Gill and mantle (*n* = 8 per treatment and tissue) were homogenized in 50 mM imidazol buffer (pH 7.2), 100 mM NaF, 5 mM EDTA, 5 mM EGTA, 15 mM 2-β mercaptoethanol, and some crystals of phenylmethylsulfonyl fluoride (PMFS) [ratio 1:5 g fresh weight (FW)/mL] with a Precellys homogenizer (2 cycles at 5,000 x g at 4°C for 15 s). Homogenates were centrifuged at 18,000 x g and 4°C for 20 min and the supernatant was used to measure PK, PEPCK, malate dehydrogenase (MDH) and LDH activities. All enzymes activities were analyzed according to the methods described by Greenway and Storey (1999) at 20°C. The absorbance at 340 nm was monitored for 10 min in a Tristar LB941 plate reader (Berthold Technologies GmbH & Co. KG, Bad Wildbad, Germany) and a molar absorbance for NADH (ε_340_ 6,220 M^−1^.cm^−1^) was used for calculations.

PK activity was measured as the oxidation of a 0.15 mM NADH solution in a 100 mM imidazole buffer (pH 7.2), 50 mM KCl, 5 mM MgCl_2_, 1 mM PEP, 2 mM ADP, 0.2% (v/v) rotenone in ethanol, and 1 U/mL LDH. Results are expressed as U PK per g FW. PEPCK was measured as the oxidation of a 0.15 mM NADH solution in a 100 mM imidazole buffer (pH 6.6), 30 mM 2-β mercaptoetanol, 5 mM PEP, 50 mM NaHCO_3_, 1.25 mM inosin-5'-diphosphate (IDP), 1 mM MnCl_2_, 2.5 U/mL MDH. Results are expressed as U PEPCK per g FW. MDH was measured as the oxidation of a 0.15 mM NADH solution in 50 mM imidazole buffer (pH 7.0), 10 mM MgCl_2_, 20 mM oxaloacetate. Results are expressed in U MDH per g FW. LDH activity was measured as the oxidation of a 0.15 mM NADH solution in 50 mM imidazole buffer (pH 7.0) after addition of 2 mM MgCl_2_, 4 mM pyruvate. Results are expressed as U LDH per g FW. PK/PEPCK and MDH/LDH ratios were calculated and used as indicator of activation of anaerobic pathways in molluscs (Grandón et al., 2008; Ivanina et al., 2016).

SOD and CAT activity were measured in mantle (*n* = 6 per treatment). Samples were homogenized in 50 mM KPi, 120 mM KCl, 0.1 mg/mL PMSF, and 1:200 Protease Inibitor Cocktail (Sigma P8340) with a ratio 1:4 w/v. Homogenates were separated, the SOD part was centrifuged at 14,000 x g for 3 min at 4°C and the supernatant was used to measure SOD activity at 20°C following Livingstone et al. (1992). The rest of the sample was treated with 0.1% Triton X-100, centrifuged at 13,000 x g for 20 min at 4°C. CAT activity was determined in the supernatant at 20°C according to the method described by Aebi (1984). Results are expressed as U per mg of protein. Proteins were measured by the method described by Bradford (1976).

GPx activity was measured in mantle (*n* = 8 per treatment except for anoxia-exposed bivalves, *n* = 6). Samples were homogenized in 20 mM Tris–HCl pH 7.6, 1 mM EDTA, 1 mM DTT with a ratio 1:4 w/v using a Precellys homogenizer (2 cycles at 5,000 x g at 4°C for 15 s). Homogenates were centrifuged at 15,000 x g for 15 min at 4°C and the supernatant was used to measure GPx activity at 20°C according to the method of Günzler and Flohe-Clairborne (1985). Results are expressed as U per mg of protein. Proteins were measured by the method described by Bradford (1976).

Caspases 3/7 activities as indicators of apoptosis intensity were measured in mantle (*n* = 6 per treatment) using the Caspase-Glow 3/7 Assay kit (Promega, USA) following the manufacturer's instructions. Samples were homogenized in lysis buffer consisting in 25 mM HEPES, 5 mM MgCl_2_·6H_2_O, 1 mM EGTA, 1 μg/mL pepstatin, 1 μg/mL leupectin, and 1 μg/mL aprotinin at a ratio 1:100 (Rivera-Ingraham et al., 2013b) using a Precellys homogenizer (2 cycles at 5,500 x g at 4°C for 20 s). Homogenates were centrifuged at 13,000 x g at 4°C for 15 min and the supernatant was used to measure luminescence using Tristar LB941 plate reader (Berthold Technologies GmbH & Co. KG, Bad Wildbad, Germany). Results are expressed as relative light units (RLU) per μg of protein × 10^4^. Proteins were quantified according to the method described by Bradford (1976).

### Succinate concentration

Mantle samples were homogenized (*n* = 10 per treatment) in 0.134 M phosphate buffer (pH 7.0) (ratio 1:5 g fresh weight (FW)/mL) using a Precellys homogenizer (2 cycles at 5,000 x g at 4°C for 15 s). Homogenates were centrifuged at 10,000 x g at 4°C for 10 min and succinate concentration was measured in mantle supernatant with a kit (Cat. No. 10176281035 Boehringer Mannheim/r-biopharm, Germany) following the manufacturer's instructions. The decrease of absorbance at 340 nm (corresponding to the consumption of NADH) was recorded in a Tristar LB941 plate reader (Berthold Technologies GmbH & Co. KG, Bad Wildbad, Germany). Results are expressed as mg succinate per g FW.

### Protein carbonyls content

Gill samples were homogenized (*n* = 8 per treatment) in 0.134 M phosphate buffer (pH 7.0) (ratio 1:5 g fresh weight (FW)/mL) using a Precellys homogenizer (2 cycles at 5,000 x g at 4°C for 15 s). Homogenates were centrifuged at 10,000 x g and 4°C for 10 min and protein carbonyl content was measured in gill supernatants using the OxiSelect Protein Carbonyl kit (Cell Biolabs Inc., San Diego, CA) following the manufacturer's instructions. Results are expressed in nmol of carbonyls per mg of protein. Protein content of the extracts was determined after Bradford (1976).

### Electron transport system activity (ETS)

ETS activity (activity of NADH dehydrogenase, i.e., complex I, and cytochrome c reductase, i.e., complex III) was measured in mantle according to Chatelain et al. (2008) and is based on the reduction of iodonitrotetrazolium (INT), INT-tetrazolium to INT-Formazan. Mantle samples (*n* = 10 per treatment) were homogenized in 20 mM TRIS buffer (pH 7.4), 1 mM EDTA, 0.1% Tween 20 [ratio 1:5 g fresh weight (FW)/mL] with a Precellys homogenizer (2 cycles at 5,000 x g at 4°C for 15 s). Homogenates were incubated in 100 mM imidazole buffer (pH 8), 3.2 mM INT, 10 mM sodium azide, 32 mM imidazole, and 0.4 mM NADH. The reduced INT was recorded in each sample using a Tristar LB941 plate reader (Berthold Technologies GmbH & Co. KG, Bad Wildbad, Germany) at 490 nm at 10°C. Results are expressed as U ETS per g FW considering a molar absorbance for INT-Formazan (ε_490_ 15.9 mM^−1^.cm^−1^) for calculations.

### Statistical analysis

Differences among treatments (normoxia, hypoxia, and anoxia) and tissues (gill and mantle) in gene expression levels were analyzed using two-way ANOVA followed by a Bonferroni's *post-hoc* test. Differences in biochemical variables among treatments were analyzed by one-way ANOVA followed by a Dunnett's *post-hoc* test since some variables could only be measured in mantle or in gill. Normality and homogeneity of variances were tested by Shapiro-Wilks'and Levene's tests, respectively (Balzarini et al., 2008). Graph Pad Prism 6 and Info Stat v. 2014 software were used for statistical analysis.

## Results

No unresponsive bivalves were observed by checking mantle and foot retraction upon mechanical stimulation at the end of the experimental exposure to normoxia, hypoxia, or anoxia.

### Metabolic response to 10 days of hypoxia and anoxia

**Figure 1:**
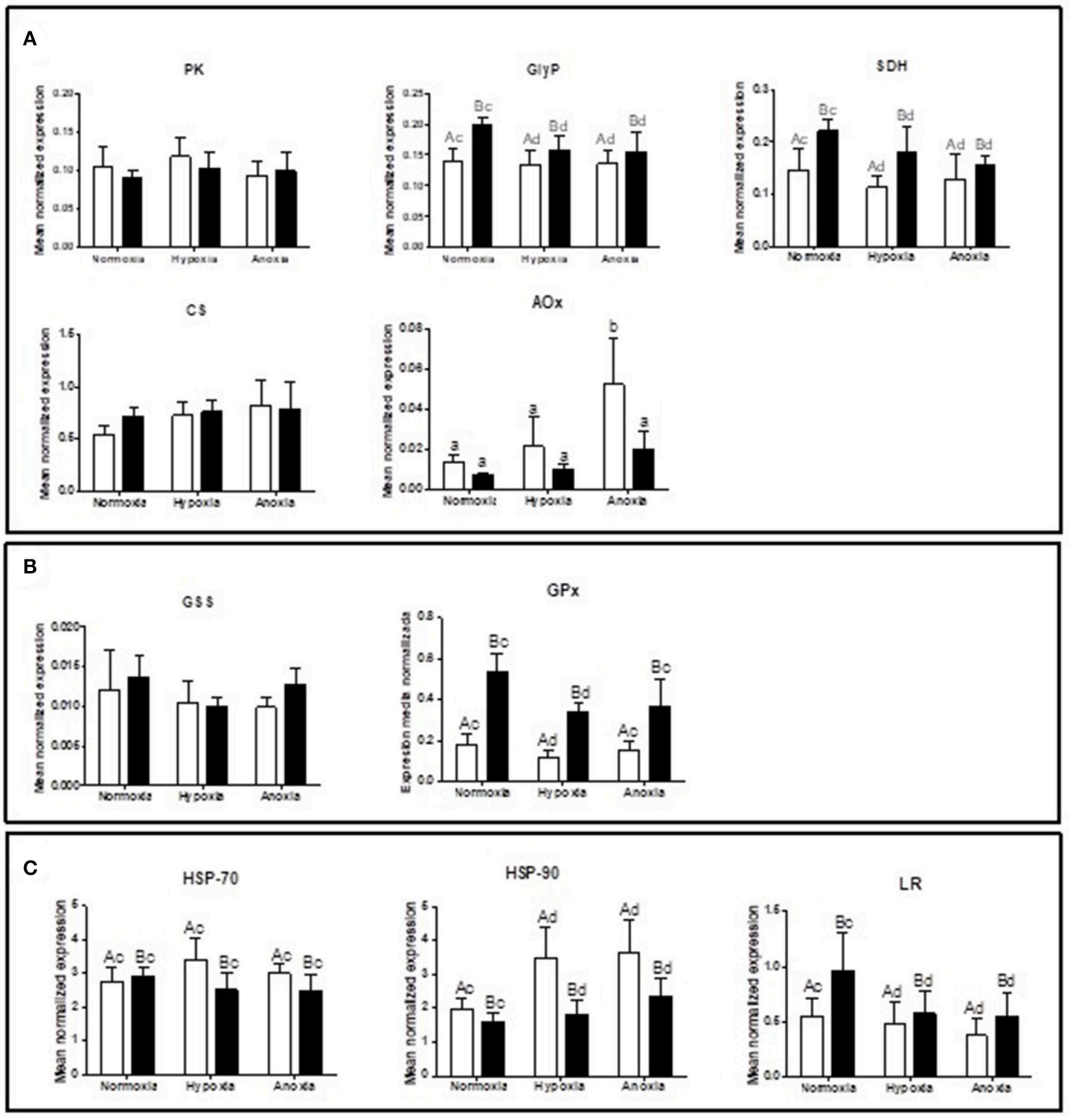
Gene expression levels of **(A)** PK (pyruvate kinase), GlyP (glycogen phosphorylase), SDH (succinate dehydrogenase), CS (citrate synthetase), and AOX (alternative oxidase); **(B)** GSS (glutathione synthetase) and GPx (glutathione peroxidase); and **(C)** HSP-70, HSP-90, and LR (laminin receptor) in gill (white bars) and mantle (black bars) of *D. chilensis* exposed to anoxia (<0.2 mg O_2_/L), hypoxia (2 mg O_2_/L), and normoxia (9 mg O_2_/L, control group), (means ± SD, *n* = 5, except for normoxia-exposed bivalves in mantle *n* = 4). Letters a and b indicate significant differences between tissues, c and d among oxygen concentration conditions (*p* < 0.05). For AOX different letters indicate significant differences (*p* < 0.05).

**Figure 2:**
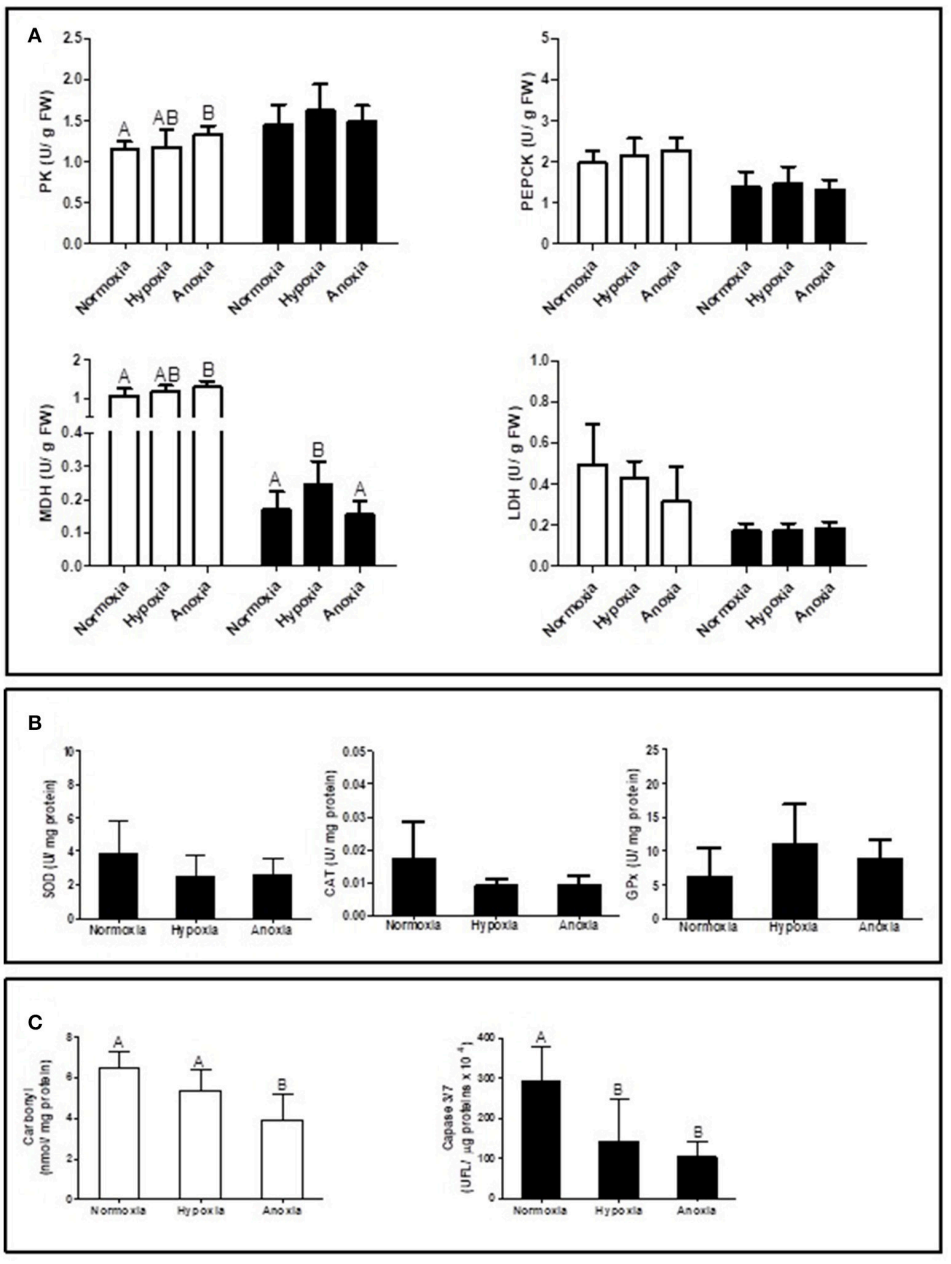
Enzyme activities of **(A)** PK (pyruvate kinase), PEPCK (phosphoenol-pyruvate-carboxykinase), MDH (malate dehydrogenase), LDH (lactate dehydrogenase) in gill (white bars) and mantle (black bars), **(B)** SOD (superoxide dismutase), CAT (catalase), and GPx (glutathione peroxidase) in mantle; and **(C)** carbonyl levels in gill and caspase 3/7 activity in mantle of *Diplodon chilensis* exposed to anoxia (<0.2 mg O_2_/L), hypoxia (2 mg O_2_/L), and normoxia (9 mg O_2_/L, control group). Different letters indicate significant differences (*p* < 0.05) (means ± SD).

Supplementary Table summarizes the statistical analysis from the gene expression study in gills and mantle. The list of analyzed genes is limited to parameters of interest based on a newly sequenced transcriptome for *D. chilensis* (Rocchetta et al., in preparation, accession numbers in Table 1). Gene expression of GlyP and of SDH were generally higher in mantle than in gill tissues and decreased significantly during 10 days of hypoxic and anoxic exposure compared to normoxic controls. Neither PK nor CS mRNA transcript levels showed significant differences between treatments or tissues (Figure 1A). In spite of the absence of change in gene transcription, the catalytic activities of PK and of MDH increased under anoxia compared to normoxia in gills (Figure 2A). Figure 2A shows in addition that mantle tissue MDH activity increased in hypoxia but not in anoxia. Neither PEPCK nor LDH activities changed in response to oxygen treatment in either tissue (Figure 2A). Concentrations of the anaerobic metabolite succinate in mantle tissue increased between normoxia (0.143 ± 0.047 mg g^−1^ FW) and hypoxia (0.267 ± 0.127 mg g^−1^ FW, *p* < 0.001, *n* = 10 in both groups) but did not differ from normoxic levels in animals exposed to anoxia (0.130 ± 0.065 mg g^−1^ FW, *n* = 10). The PK/PEPCK ratio did not show differences between treatments in gill or mantle tissue whereas the MDH/LDH ratio showed significant differences only in gills with higher values under anoxia compared to normoxic controls (MDH/LDH normoxia 2.46 ± 1.09, hypoxia 2.87 ± 0.72, and anoxia 3.89 ± 1.05). AOX expression and ETS activities showed the strongest change of all measured parameters with significant interaction between oxygen concentration and tissue. In gills of animals from the anoxia treatment AOX transcript levels were four-fold higher than in the normoxic controls (*p* < 0.05, Figure 1A). A similar trend in AOX gene transcription was observed in mantle tissue but did not reach significance. Mitochondrial ETS activity could only be measured in mantle tissue (gill samples being consumed in other analyses) and was reduced by one-third in bivalves exposed to anoxia compared to the normoxic controls (normoxia 0.062 ± 0.0192, hypoxia 0.059 ± 0.012, and anoxia 0.041 ± 0.014, *p* < 0.01).

### Stress response to 10 days of hypoxia and anoxia

GPx expression levels were higher under normoxia than under hypoxia in gill and in mantle, while GSS expression did not show differences across treatments (Figure 1B). Both heat shock genes were more highly expressed in gills than in mantle with no interaction between oxygen concentration and tissue (Figure 1C). HSP90 expression levels were higher under oxygen deplete conditions than under normoxia (Figure 1C). LR transcript levels decreased between normoxic controls and hypoxic/anoxic incubations in both tissues (Figure 1C) and were consistently higher in mantle than in gills.

Antioxidant activity in mantle (SOD, CAT, and GPx) did not show significant differences between treatments, but a trend to decrease under hypoxia and anoxia compared to normoxic controls was observed for SOD and CAT activities (Figure 2B).

Oxidative damage to proteins in gill tissue was reduced by one third in anoxia exposed bivalves compared to normoxic controls (Figure 2C). A similar decline was observed for mantle caspase 3/7 activity. Normoxic control tissues had significantly higher caspase activity than tissue samples from both hypoxia and anoxia exposed individuals (Figure 2C).

## Discussion

Marine and freshwater clams with benthic lifestyle are interesting physiological models owing to their extreme hypoxia/anoxia tolerance. The freshwater bivalve *D. chilensis* is an excellent example, and we studied its tiered adaptive response to 10 days of low and no oxygen availability, including changes in the mitochondrial respiratory system, use of energetic pathways, and the cellular stress response.

### Coordinated response to oxygen deficiency in *D. chilensis* mitochondria

**Figure 3:**
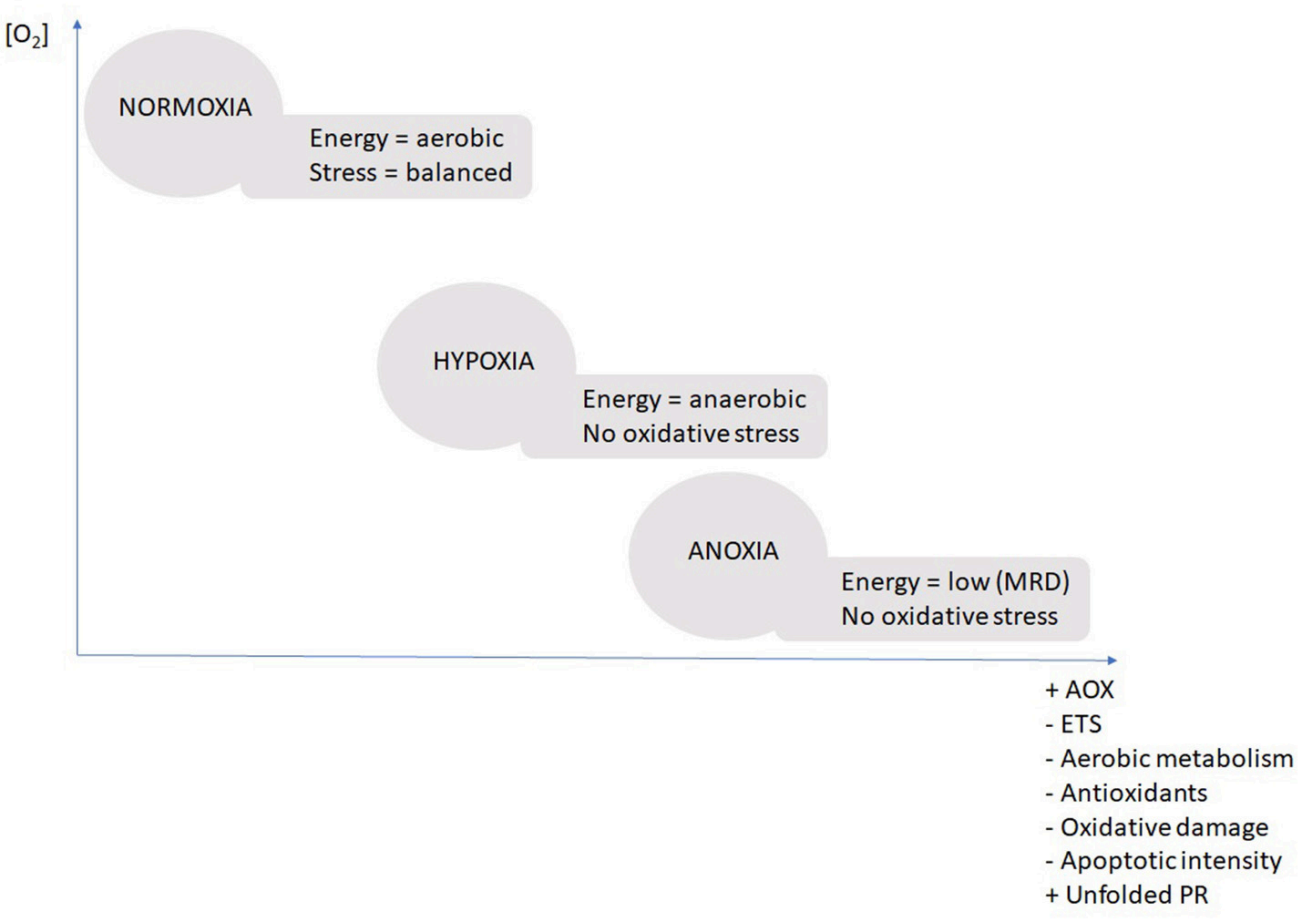
Schematic diagram summarizing the major physiological mechanisms of adaptation to different oxygenation scenarios. Scenarios “normoxic,” “hypoxic,” and “anoxic” are based on evidence from the present study. AOX, alternative oxidase; ETS, electron transport system of complex I and III; unfolded PR, unfolded protein response; MRD, metabolic rate depression.

The diagram below summarizes the stepwise response throughout the three treatment conditions (Figure 3). The central response was the stepwise reduction of ETS activity in combination with the up-regulation of the AOX in both tissues. In hypoxic conditions the bivalves initiate AOX upregulation (non-significant but detectable), but it takes exposure to nearly complete anoxia (2 and 0.2 mg O_2_ L^−1^) for the AOX pathway to become the dominant route for the electrons.

Both, the reduced ETS activity and the induction of AOX transcription (and translation) are necessary, because the AOX is assumed to have lower oxygen affinity than residual cytochrome-c oxidase (Tschischka et al., 2000: defined for two oxyconforming marine invertebrates, the polychaete *Nereis diversicolor* and the clam *A. islandica*). Under *in-situ* conditions in a hypoxic/anoxic lake and potentially sulfidic sediments, animals can experience shutdown of cytochrome-c oxidase activity through H_2_S inhibition, which highlights the necessity to shunt electrons through the alternative pathway. Hence, the AOX also has an important role to play during reoxygenation which consists in maintaining electron flux and mitochondrial membrane potential as long as it takes to oxidize resident sulfide to non-toxic thiosulfate and re-initiate the energetically more efficient classical ETS.

It is intriguing that the AOX response was much more pronounced in gills than in mantle tissue. This is the organ through which H_2_S would first enter the animals and where its effect on cytochrome-c oxidase would be maximal. Moreover, gills store no appreciable glycogen reserves and instead reduce cellular energy demand (ciliary beat frequency and investments into stress defense) and coordinate the shutdown of ETS activity and ATP production to conserve mitochondrial membrane potential under low oxygen.

Enhanced expression of AOX in hypoxia has been shown only once before by Sussarellu et al. (2012) for *C. gigas* gills. Recently, Liu and Guo (2017) reported a novel AOX derived from alternative splicing of duplicated exon in the oyster *Crassostrea virginica*. Expression of both splice variants was highest in gills compared to all other tissues and one of these AOX variants was increasingly expressed during air exposure which presumably caused anoxia in shell water. Contrary the other splice variant was always the dominant transcript under normoxic conditions. It appears possible that the “AOX system” is a mechanism that controls cellular oxygen levels at both ends of the oxygenation spectrum (too high and too low) in bivalves experiencing highly fluctuating shell water and tissue oxygenation levels (see Abele and Philipp, 2013). The present study highlights for the first time the complete mechanism of shunting electrons between the classical and the branching AOX pathway within the overall context of MRD in a hypoxia tolerant clam.

Mitochondrial anaerobic pathways were stimulated under anoxia in gills as indicated by the increase in the MDH/LDH. This ratio was already established as a marker indicating environmental anaerobiosis in *D. chilensis* (see Grandón et al., 2008). Prolonged environmental oxygen deficiency usually leads to diversion of the glycolytic flux to the more efficient mitochondrial pathway, a strategy of hypoxia tolerant species to minimize depletion of glycogen stores. In the present case this is reflected in the down-regulation of GlyP expression after 10 days of hypoxic and anoxic exposure. Contrary we saw no obvious regulation of PK transcription and measurements of enzyme activities gave no further indication of an activation of PEPCK or deactivation of PK after 10 days of oxygen deplete conditions. Kinetic analyses of PK affinity for PEP across groups might clarify these results.

Slightly induced PK activity in gills might indicate activation of the aspartate consuming/alanine producing transamination of pyruvate in anoxia exposed *D. chilensis*. This pathway couples glucose (glycogen) fermentation to amino acid (aspartate) catabolism in marine bivalves during prolonged anoxia (see De Zwaan and Wijisman, 1976; Collicutt and Hochachka, 1977; Zurburg and Kluytmans, 1980; Issani et al., 1995). In the absence of any further metabolite measurements (alanine and aspartate) we can, however, not make a clear statement toward activation of this pathway in the freshwater bivalves. Contrary to gills, there is no sign of PK activation in mantle tissue, and instead succinate accumulates, which indicates mitochondrial anaerobiosis to support the energetic state of mantle tissue under hypoxic conditions. As MRD intensifies in anoxia, mantle succinate diminishes, most likely due to conversion to short chained organic acids (propionate and acetate) for slightly better anaerobic ATP gain and CO_2_ binding (Hochachka et al., 1996; Larade and Storey, 2002). In both marine and freshwater bivalves, propionate is eventually excreted into the surrounding medium after several days in anoxia to limit tissue acidification (Holwerda and Veenhof, 1984; van den Thillart and de Vries, 1985; Issani et al., 1995). All these constraints to energetic efficiency in anoxia lead to a reduction of energy consuming cellular processes such as apoptosis (caspase activity) and the unfolded protein response in mantle tissue.

Our analysis highlights the tissue specific response to environmental oxygen shortage: *D. chilensis* gills, the main respiratory tissues, rely on the AOX pathway to support partial electron transport in order to stabilize mitochondrial membrane potential during metabolic shutdown in anoxia. This is of pivotal importance for mitochondrial integrity in hypoxia tolerant species even if no substantial ATP production occurs. In mantle tissue, however, anaerobic glycolysis is activated in hypoxia before metabolic shutdown reduces the overall energy demand in anoxia.

It is interesting to note that Ivanina et al. (2016) measured an increase of mitochondrial oxygen consumption capacity (termed “ETS” in their paper) in isolated gill mitochondria during hypoxic exposure of the marine hard clam *Mercenaria mercenaria*, which is of comparable hypoxia tolerance as *D. chilensis*. Note that hypoxic incubation in that paper lasted only 18 h and therefore shows the acute rather than the adaptive, long-term response to hypoxia. At the same time, phosphorylation capacity (PHOS in Ivanina et al., 2016) decreased to one third of normoxic values in hypoxia, matching the shortcut of complex III and IV in the mitochondria. While the authors attributed the intensified oxygen consuming capacities in the “hypoxic” mitochondria (assayed under fully oxic conditions) to mechanisms such as reversible phosphorylation of ETS compounds, an alternative mechanism to be explored in *Mercenaria* would indeed be on-switch of the AOX pathway.

### Response of stress proteins during anoxic transgression in *D. chilensis*

As summarized in Figure 3 there were no signs of enhanced oxidative stress during anoxic transgression (normoxia > hypoxia > anoxia) in *D. chilensis* and much to the contrary oxidative stress parameters (protein carbonyls and apoptotic intensities) decreased stepwise from normoxia toward anoxia. Furthermore, there was no indication of antioxidant enzyme induction at either transcript (GPx and GSS) or enzyme activity level (SOD or CAT). Indeed, the trend to diminish SOD and CAT activity indicates a slowdown of mitochondrial ROS production as oxygen levels declined. The transcript levels (GPx and GSS) were maintained stable under anoxia indicating that a basal protection against H_2_O_2_ accumulation is kept up. This is important basal protection as H_2_O_2_ is always present in bivalve hemolymph and tissues where it serves as an antimicrobial defense agent. Within the cells, H_2_O_2_ levels need permanent control to prevent hydroxyl radical formation through Fenton chemistry, the more so when CAT levels are low in hypoxia/anoxia. Significant reduction of antioxidant defenses was previously observed in another hypoxia tolerant species, the gastropod *Littorina littorea* (Pannunzio and Storey, 1998: SOD, CAT, GR, GSS, GPx following 6 days of anoxia), whereas anoxic exposure had no effect on antioxidants in *A. islandica* (Strahl et al., 2011a: SOD, CAT). David et al. (2005) reported changes of gene expression patterns in hypoxia exposed oysters (*C. gigas*), where GPx mRNA was upregulated and SOD mRNA was among the downregulated stress enzymes following 24 days of hypoxia and thus resembling the basal pattern we observed in *D. chilensis* with SOD decline during phases of reduced respiratory rates (or MRD) and GPx activity constant, controlling levels of H_2_O_2_ in hypoxia.

Of all stress proteins we analyzed, only HSP90 an important cellular chaperone in bivalves was substantially upregulated in hypoxia/anoxia in the gills, indicating the necessity for an unfolded protein response. HSP90 has been shown to interact with cytoskeletal elements during episodic cell stress, especially heat and chemical stress (summarized by Fabbri et al., 2008), and ours seems to be the first report of its induction during oxygen deficiency. Both chaperones HSP70 and 90 have been implicated to interfere with apoptotic pathways, e.g., in human tumors, and recently overexpression of HSP90 and its interaction with the p53 tumor suppressor protein (or its mutant variants) has been shown to enhance intensity of hemolymphatic neoplasia in cockles (Díaz et al., 2011). Hence, overexpression of HSP90 in combination with the decrease in caspase 3/7 activity and LR expression points toward suppression of apoptotic cells death in hypoxia/anoxia exposed gill tissues. As cellular turnover including apoptosis and proliferation is rather active in gills compared to other organs of bivalves (Strahl and Abele, 2010) this may be another energy saving protective mechanism during periods of reduced ATP availability.

Our findings show a strategy in *D. chilensis* to conserve energy through a reduction of metabolic rate under long-term exposure to oxygen depletion. The stepwise response to hypoxic condition induces ETS reduction in combination with an up-regulation of the AOX which becomes the main electron transport route under anoxic conditions. When oxygen is completely absent no oxidative stress occurs in bivalves (i.e., no signs of antioxidant induction and decrease in protein carbonyls and apoptotic intensities in the transgression from normoxia to anoxia). Up-regulation of HSP90 ensures protection of mitochondrial integrity during periods of oxygen depletion. These variables are proposed as useful biomarkers of oxygen deficiency to be measure in bivalve gills in the Patagonian lakes.

## Data accessibility

DNA sequences: GenBank accession numbers in Table 1.

## Author contributions

MY, IR, and DA: designed the project; MY: performed research; SS, MR, and IR: contributed laboratory work; CL: collected field samples; CH: contributed to analyze molecular data; MY: wrote the manuscript; DA, CH, CL, and IR: contributed to writing with input from all authors. DA, CH, IR, and MR: contributed funding to the project.

### Conflict of interest statement

The authors declare that the research was conducted in the absence of any commercial or financial relationships that could be construed as a potential conflict of interest.
