# Corrigendum: Inducing the Alternative Oxidase Forms Part of the Molecular Strategy of Anoxic Survival in Freshwater Bivalves

**DOI:** 10.3389/fphys.2018.00330

**Published:** 2018-03-27

**Authors:** Maria S. Yusseppone, Iara Rocchetta, Sebastian E. Sabatini, Carlos M. Luquet, Maria del Carmen Ríos de Molina, Christoph Held, Doris Abele

**Affiliations:** ^1^Laboratorio de Enzimología, Estrés y Metabolismo, INQUIBICEN, Departamento de Química Biológica, Facultad de Ciencias Exactas y Naturales, Universidad de Buenos Aires, Consejo Nacional de Investigaciones Científicas y Técnicas, Buenos Aires, Argentina; ^2^Laboratorio de Ecotoxicología Acuática, INIBIOMA, Universidad Nacional del Comahue, Consejo Nacional de Investigaciones Científicas y Técnicas, Junín de los Andes, Argentina; ^3^Department of Functional Ecology, Alfred Wegener Institute, Helmholtz Centre for Polar and Marine Research, Bremerhaven, Germany

**Keywords:** hypoxia, oxidative stress, alternative oxidase, mitochondrial electron transport, anaerobiosis, *Diplodon chilensis*

In the original article, we neglected to add Dr. Doris Abele as correspondence author. Thus, in the author list her name should be also indicated with an asterisk and added in the list of correspondence authors. The authors apologize for the mistake. This error does not change the scientific conclusions of the article in any way.

The original article has been updated.

## Conflict of interest statement

The authors declare that the research was conducted in the absence of any commercial or financial relationships that could be construed as a potential conflict of interest.

