# Supplementary material for: Inducing the Alternative Oxidase Forms Part of the Molecular Strategy of Anoxic Survival in Freshwater Bivalves
Source: Front Physiol. 2018 Feb 23;9:100. doi: 10.3389/fphys.2018.00100 (PMC5829090; doi:10.3389/fphys.2018.00100)
Supplement: Supplementary file 1 [file Table1.docx]

**Supplementary Table 1**

Summary of statistical analysis of gene expression in *D. chilensis*. PK pyruvate kinase, GlyP: glycogen phosphorylase, SDH: succinate dehydrogenase, CS: citrate synthetase, AOX: alternative oxidase, GSS: glutathione synthetase, GPx: glutathione peroxidase, HSP: heat shock protein (70 and 90), LR: laminin receptor.

| Gene | P value [O_2_] | P value tissue | P value [O_2_] _*_ tissue |
| --- | --- | --- | --- |
| PK | 0.3026 | 0.3399 | 0.4901 |
| GlyP | 0.047 | 0.0007 | 0.1401 |
| SDH | 0.0442 | 0.0005 | 0.3496 |
| CS | 0.0972 | 0.3714 | 0.4401 |
| AOX | ----- | ----- | 0.0487 |
| GSS | 0.2237 | 0.1584 | 0.4221 |
| GPx | <0.0055 | <0.0001 | 0.1051 |
| HSP70 | 0.6058 | 0.0191 | 0.066 |
| HSP90 | 0.0016 | 0.0001 | 0.113 |
| LR | 0.0117 | 0.0066 | 0.2156 |
